# A Meaty Problem: Radiological Perspective on an Unusual Rectal Foreign Body

**DOI:** 10.7759/cureus.95762

**Published:** 2025-10-30

**Authors:** Ibrahim Feyyaz Naldemir

**Affiliations:** 1 Radiology, Duzce University, Düzce, TUR

**Keywords:** foreign body insertion, imaging in a rectal foreign body, rectal foreign body, risky sexual experimentation, unusual rectal foreign objects

## Abstract

Rectal foreign bodies (RFBs) are a rare but notable cause of presentation. They are usually associated with sexual gratification, accidents, or psychiatric conditions. This case report discusses the diagnosis and management of a rare food-borne RFB. A 67-year-old man presented with perianal pain and a feeling of fullness in the rectum. He stated that he was under the influence of alcohol and did not remember the events. Physical examination and radiologic imaging revealed a 28-cm-long baton sausage with metallic material at the tip in the rectum. The foreign body was removed by colonoscopy without complications. RFB cases usually involve males and are associated with sexual objects. CT plays a critical role in the diagnosis and evaluation of complications, while endoscopic methods usually provide effective treatment. With proper imaging and endoscopic management, successful outcomes can be achieved without the need for surgical intervention. A multidisciplinary approach is important to improve patient outcomes.

## Introduction

Rectal foreign bodies (RFBs) are a rare but notable cause of emergency department visits. These cases often arise from intentional acts and can occur for a variety of reasons, including sexual gratification, psychiatric disorders, or accidental ingestion [1]. Oftentimes, patients delay seeking treatment due to embarrassment or reluctance to seek medical help, which can increase the risk of complications [2].

Radiologic imaging plays a critical role in establishing the diagnosis and treatment plan in these cases. Although direct radiography is usually the first choice for the detection of RFBs, advanced imaging modalities such as computed tomography (CT) may be required for detailed anatomic evaluation and determination of complications [3].

This case report discusses the evaluation, diagnostic process, and treatment of a patient who had a "sausage" lodged in his rectum. Food-related RFBs are rarely reported in the literature, and this case provides valuable insights for healthcare professionals managing similar conditions in similar cases.

## Case presentation

A 67-year-old male patient presented to the emergency department complaining of perianal pain. The patient stated that he drank a lot of alcohol alone at night and did not remember anything. When he awoke in the morning, he reported severe pain and redness in the pelvic and anal areas, a sensation of fullness in the rectum and anus, and swelling in the suprapubic area. On further questioning, although he could not remember anything, he stated that something may have been inserted into his anus. He stated that he was not accompanied by anyone when he drank alcohol at night. The patient had no comorbidities other than hypertension and no diagnosed psychiatric illness.

A physical examination of the patient revealed redness of the perianal skin. The external anal sphincter tone was decreased, and a rectal palpation revealed the presence of a small, hard object distal to the rectum and a larger, soft material just above it. In addition, a palpable and tender swelling was observed on the abdominal wall at the suprapubic level. Consequently, the patient underwent an abdominal roentgenogram, which yielded a preliminary diagnosis of a foreign body in the rectum. This was followed by an abdominal CT scan with contrast to visualize any potential complications.

**Figure 1:**
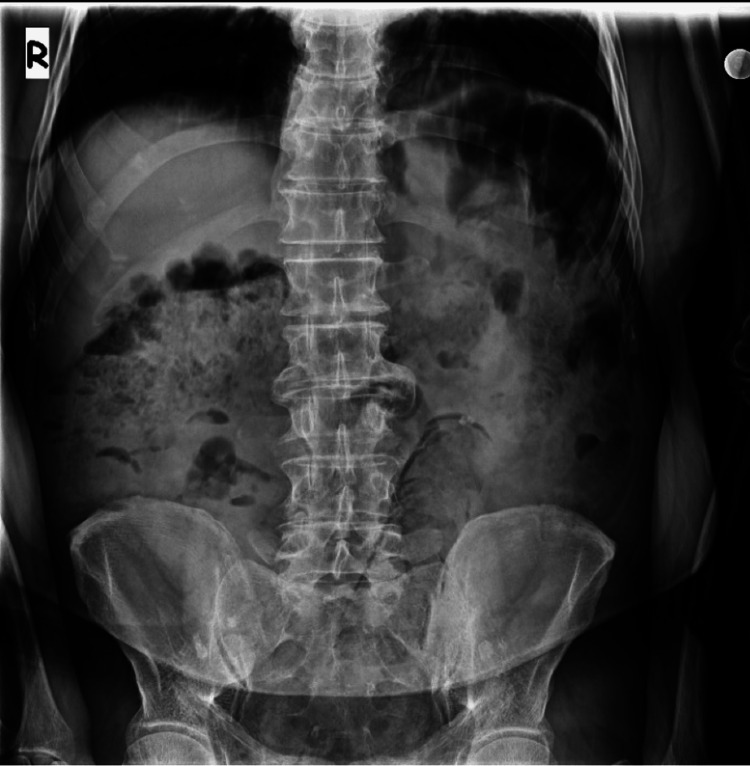
The presence of a tubular lesion with radiolucent borders in the pelvic region is indicative of a foreign body.

**Figure 2:**
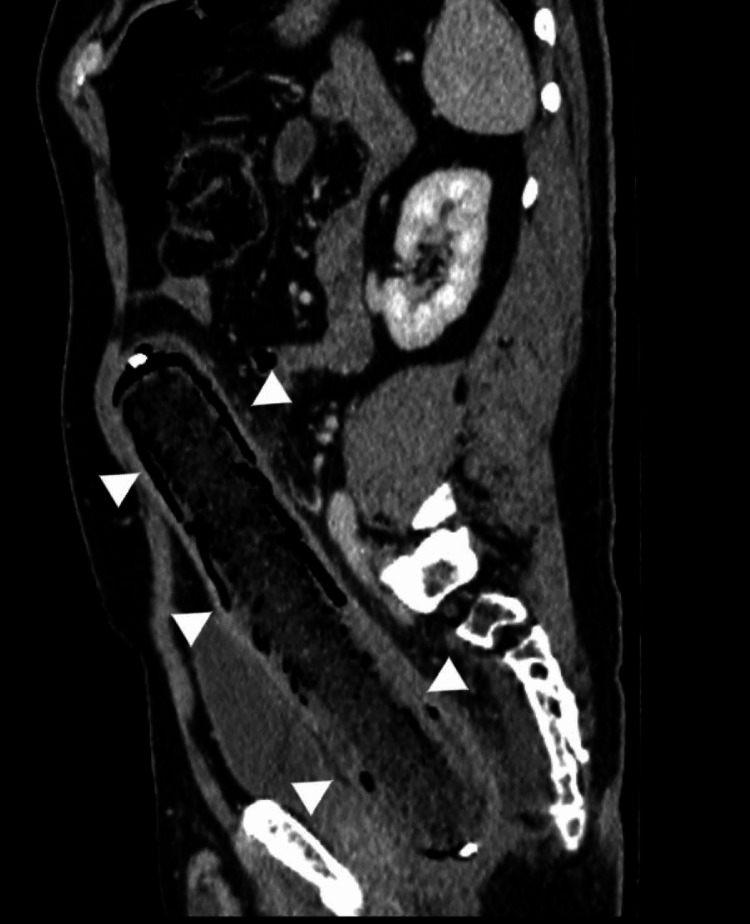
The oblique sagittal CT image reveals the presence of a baton sausage, which originates at the distal rectum and extends through the sigmoid colon (arrow heads). Note the bulging of the abdominal wall.

**Figure 3:**
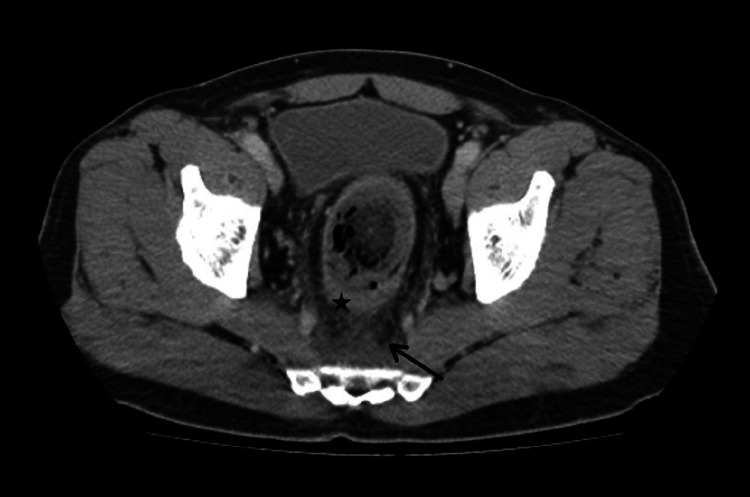
Axial CT image reveals diffuse thickening of the rectal wall (asterix), as well as edematous contamination of perirectal and presacral adipose tissue (arrow).

A radiographic analysis revealed the presence of a tubular structure originating from the pelvic region and extending into the left abdominal cavity (Figure 1). This structure exhibited a linear radiolucent appearance along its periphery, accompanied by a focal opaque focus in its apex. CT imaging revealed a foreign body measuring 28 centimeters in length, extending up to the level of the sigmoid colon in the rectum (Figure 2). The foreign body exhibited a metallic component at the proximal and distal parts, with predominantly fat-like characteristics. Diffuse minimal thickening of the rectal wall, contamination of the surrounding fatty tissue, and a small amount of free fluid in the presacral region were observed (Figure 3). No free air value suggestive of perforation was observed. The object under investigation did not correspond to any documented instance of a sex toy or foreign body. It was hypothesized that the object was a foodstuff due to its fat density. When informed about the presence of a foreign body in his rectum, the patient mentioned having a baton sausage at home and speculated that it might be the object, although he could not recall how it ended up there. Consequently, a colonoscopy was performed, which revealed a foreign body consistent with a baton sausage, featuring metallic material at the distal end of the rectum and a patent outer container, aligning with the patient's description. The foreign body was successfully extracted in its entirety with the use of forceps. The patient was monitored for a subsequent day and was discharged without complications.

## Discussion

Although RFBs are uncommon [4], they are among the interesting and sometimes complex cases that may be encountered in emergency departments. A review of the extant literature reveals that such cases are most often associated with accidents, sexual activity, or psychiatric conditions [2]. In one study, it was reported that the majority of RFB cases were seen in men and were frequently associated with the use of sexual objects [4]. However, food-based foreign bodies are rarely reported in the literature. This case makes an important contribution to the literature in terms of the diagnosis and treatment of a food-based foreign body in the rectal region.

In the diagnosis of RFB, the patient's history is often limited. The patient may be reluctant to share information due to feelings of embarrassment or social pressure, which can complicate the diagnostic process [5]. Furthermore, as evidenced in this case, the patient's memory impairment following alcohol consumption can impede the diagnostic process. In such cases, healthcare professionals must prioritize the establishment of a trusting relationship with their patients by demonstrating an empathic approach, ensuring the accuracy of diagnosis, and the development of an effective treatment plan.

Imaging methods are indispensable in the diagnostic process. While radiography is generally preferred as the initial method, computed tomography (CT) provides important information in the evaluation of the exact localization of the foreign body, its dimensions, and possible complications [4]. CT has been shown to have a high sensitivity in the early detection of complications such as perforation and fistula development [6]. In the present case, the CT scan provided detailed information regarding the characteristics and local effects of the foreign body in the rectum, thereby facilitating the diagnostic process.

Minimally invasive methods are generally preferred in the management of RFBs. According to the literature, approximately 80-90% of foreign bodies can be effectively removed by endoscopic methods. However, surgical intervention may be necessary in cases of complications such as perforation, peritonitis, or severe rectal bleeding [7]. In this case, the successful removal of the foreign body with the use of a colonoscopy once again demonstrates the efficacy of minimally invasive methods. However, the removal of large or irregularly shaped foreign bodies necessitates thorough advance planning and, when deemed essential, laparotomy due to the potential for technical challenges [1].

The complication rates associated with RFBs may be subject to variation based on factors such as size, shape, removal time, and the methods employed during the procedure. The most frequently reported complications include rectal bleeding, perforation, sepsis, and rectal stenosis [7]. The absence of perforation and bleeding findings in the present case, with the exception of contamination of the mesorectal fatty tissue and thickening of the rectal wall, underscores the significance of early diagnosis and appropriate intervention. The role of psychiatric and social factors in such cases has also been a subject of frequent discussion in the literature. According to O'Farrell et al., 37.5% of patients presenting with RFBs had an underlying psychiatric disorder [8]. However, in this case, the absence of a documented psychiatric disorder and the occurrence of the event under the influence of alcohol suggest that such cases should be approached through a multidimensional perspective.

## Conclusions

The present case offers a valuable contribution to the existing knowledge by shedding light on a rare subgroup of RFBs. The management of a food-based foreign body, which is rarely reported in the literature, reveals the importance of a multidisciplinary approach in the diagnostic process. Imaging modalities such as direct radiography and CT play a critical role in establishing a definitive diagnosis and formulating an appropriate treatment plan. Moreover, the successful utilization of endoscopic methods has been shown to expedite the recovery process for patients by diminishing the necessity for surgical intervention. It is imperative for healthcare professionals to consider not only physical but also psychosocial factors in such cases, both in terms of patient satisfaction and clinical outcomes.
